# Current status of public health awareness and roles within medical institutions in eastern China

**DOI:** 10.3389/fpubh.2025.1546507

**Published:** 2025-01-14

**Authors:** Yuchen Wu, Chuanfeng Zhang, Quan She, Wei Zheng, Kai Lu, Furu Wang

**Affiliations:** ^1^Jiangsu Provincial Center for Disease Control and Prevention, Nanjing, China; ^2^The Affiliated Hospital of Xuzhou Medical University, Xuzhou, China; ^3^Kunshan Municipal Centers for Disease Control and Prevention, Kunshan, China; ^4^Key Laboratory of Modern Toxicology, School of Public Health, Nanjing Medical University, Nanjing, China

**Keywords:** public health, integration of medicine and prevention, awareness, functions, policy

## Abstract

**Objective:**

To examine the current landscape of public health functions and the level of cognition among medical staff across various medical institutions in Jiangsu Province, ultimately aiming to provide a theoretical framework for constructing a hospital public health work model under the integrated medical and defense system.

**Methods:**

A mixed-methods approach involving questionnaires and semi-structured interviews was employed to assess the establishment and operational status of public health departments within medical institutions, staffing scenarios, comprehension of public health tasks, and the degree of emphasis placed on them.

**Results:**

Our findings reveal that 70.6% of medical institutions possess standalone public health departments, with a median of 5 full-time public health personnel, predominantly from the nursing profession. Significant disparities were observed in the demographics, educational backgrounds, majors, job classifications, and titles of public health personnel across different medical institution levels. Additionally, 68.4% of institutions have formed public health management committees, and 77.2% shoulder ≥10 public health responsibilities. Notably, 99.2% of medical staff endorse the importance of public health work within medical institutions, and 98.3% advocate for the support of disease control agencies. Interviews further unveiled a predominantly positive stance among medical institution leaders toward the integration of public health and medical prevention.

**Conclusion:**

Medical institutions of Jiangsu Province necessitate enhanced public health staffing, improved organizational structure and responsibility divisions for public health work, and a boost in knowledge levels to effectively carry out public health responsibilities. Furthermore, policy support and multi-stakeholder engagement are crucial to mobilize hospitals to take proactive initiatives.

## Introduction

The cause of public health is intrinsically linked to the life safety and well-being of the populace, as well as the social stability and economic development of a nation or region (1). Since the founding of the People’s Republic of China, our country has placed significant emphasis on the development and improvement of the medical prevention system. In 1949, at the early stages of the People’s Republic, infectious diseases and endemic illnesses posed a serious threat to public health. Medical and healthcare resources were scarce and unevenly distributed between urban and rural areas. In response, China launched the “Patriotic Health Campaign,” which mobilized the masses extensively and implemented a disease control model prioritizing prevention. Notably, this campaign significantly strengthened the availability of primary healthcare services in rural areas. Since 1979, China has consistently adhered to the policy of prioritizing prevention. However, market-oriented reforms have impacted preventive healthcare, leading to more pronounced issues of medical separation. The outbreak of SARS in 2003 highlighted the lack of coordination between the medical system and the public health system, with the separation between medical and preventive systems becoming a major obstacle to the high-quality development of China’s healthcare system. These institutions shoulder public health responsibilities such as the prevention and treatment of infectious diseases, the management of chronic non-communicable diseases, and health education and promotion. Statistical data indicate that medical institutions in China provide 40 to 50% of public health services (1), and 80 to 90% of primary infectious disease information originates from these institutions (2, 3).

Currently, the operational sustainability of China’s medical institutions primarily depends on their medical service revenue, which has led to a dilution of public welfare in the execution of public health functions. This situation is characterized by multiple leadership structures, fragmented management processes, and a pronounced emphasis on curative healthcare at the expense of preventive measures. The establishment of public health departments within medical institutions serves as a robust foundation for enhancing the efficiency and coordination of public health and medical services (4–6). In Jiangsu Province, the creation of public health departments across all levels of medical institutions has been initiated, although the public health functions and scope of operations vary among different levels of institutions.

This study examines the public health awareness, functions, authorities, and operational status of medical institutions at different levels, from a three-tier perspective. It identifies key issues and influencing factors in the process of medical institutions fulfilling their public health roles, providing a theoretical basis for the research on innovative medical and preventive coordination systems and mechanisms, as well as the development of relevant policies for the integration of medical and preventive services.

## Materials and methods

### Data sources

A multi-stage sampling method was employed to select medical institutions of various levels and types across 13 districts in Jiangsu Province, encompassing public hospitals, township health centers, community health service centers, and private hospitals. At least 10% of the total workforce from each selected institution was surveyed, with professional categories including clinicians, public health physicians, nurses, medical technologists, health management personnel, and other staff members.

### Survey content

The study employed a mixed-methods approach, combining a questionnaire survey with semi-structured in-depth interviews. All levels of medical institutions within the province are included, with full coverage of provincial-level medical institutions. Specifically, each district and city has selected 10 or more secondary-level and above public hospitals. Additionally, from all counties and townships within the district, no fewer than two hospitals or community health service centers have been chosen. Where conditions allow, one to two social medical institutions within the jurisdiction have also been included. Surveys were conducted concurrently in these institutions. Participation in the survey comprised not less than 10% of the total staff in each medical institution, including clinicians, public health doctors, nursing staff, medical technicians, health management personnel, and other relevant personnel. The questionnaire covered three main areas: (1) basic information about medical institutions; (2) the current state of public health practitioners’ work functions; (3) non-public health practitioners’ understanding of the current state of public health functions. Semi-structured in-depth interviews were conducted with the heads of medical institutions and the heads of public health management departments to gain insights into the significance, current status, existing challenges, and recommendations for improving public health work in hospitals, thereby collecting and analyzing deeper-level data on the variables.

### Statistical analysis

The data were entered using software EpiData version 3.1 and data organization and cleaning were accomplished to form a database, followed by the researchers conducted a quality check of the gathered data in the field. Data analysis was performed with SPSS version 24.0 (IBM, SPSS, United States). Categorical variables were expressed as proportions and percentages. Chi-square and Fisher’s exact tests were applied as appropriate. For quantitative variables, normality was examined, and results were expressed using the mean ( $\bar{x}$ ), median (*M*), standard deviation (*SD*) and interquartile range (*IQR*). Comparisons were performed using the t-test, Mann–Whitney U, Kruskal-Wallis, and ANOVA tests. A *p*-value <0.05 was considered significant; all significance tests were two-tailed.

### SWOT analysis

Additionally, we utilize a SWOT (strengths, weaknesses, opportunities, and threats) analysis to highlight the implementation of public health programs within medical institutions. The SWOT analysis is a time-honored tool for formulating organizational strategy. SWOT analysis requires a candid assessment of organizational resources (strengths and weaknesses) in the context of external realities (opportunities and threats) (7). For the purposes of this analysis, the terminology of SWOT is adapted to reflect advantages (strengths), disadvantages (weaknesses), ongoing challenges (threats), and opportunities. This study gathered information on the implementation of public health programs by medical institutions through semi-structured interviews, and identified all strengths, weaknesses, opportunities, and threats of the research subjects. Subsequently, the study employed systems analysis theory and other methods to analyze the data and draw conclusions, and the results will be presented in a table.

## Results

### The establishment of public health departments in medical institutions

A total of 250 medical institutions were included in this survey, comprising 91 (36.4%) tertiary hospitals, 37 (14.8%) secondary hospitals, and 122 (48.8%) primary and below medical institutions. Among these, 224 (89.6%) were public medical institutions, and 227 (90.8%) were general hospitals. According to the survey on the establishment of public health departments, 190 (76.0%) have been independently established, including 90 (47.4%) tertiary hospitals, 30 (15.8%) secondary hospitals, and 70 (36.8%) primary or lower medical institutions. Of these 190 institutions, 124 (65.3%) established their public health departments after 2021, yet 113 (59.5%) lack dedicated funding for these departments (for detailed information, refer to Table 1).

**Table 1:** Basic information of the medical institutions.

Characteristics	Hospitals of grade II or above	Hospitals of sub-grade II	Total	*p*-value
Region				0.233
South Jiangsu	53 (41.4)	54 (44.3)	107 (42.8)	
Central Jiangsu	32 (25.0)	20 (16.4)	52 (20.8)	
Northern Jiangsu	43 (33.6)	48 (39.3)	91 (36.4)	
Ownership				*0.003*
Public	122 (95.3)	102 (83.6)	224 (89.6)	
Non-public	6 (4.7)	20 (16.4)	26 (10.4)	
Scope				*<0.001*
General hospital	106 (82.8)	121 (99.2)	227 (90.8)	
Specialized hospital	22 (17.2)	1 (0.8)	23 (9.2)	
Independent public health department				*<0.001*
Yes	120 (93.8)	70 (57.4)	190 (76.0)	
No	8 (6.3)	52 (42.6)	60 (24.0)	
Establishment time of public health department				*<0.001*
Before 2021	10 (8.3)	56 (80.0)	66 (34.7)	
After 2021	110 (91.7)	14 (20.0)	124 (65.3)	
Independent working expenses				*0.004*
Yes	39 (32.5)	38 (54.3)	77 (40.5)	
No	81 (67.5)	32 (45.7)	113 (59.5)	

### Public health-related staffing

According to the survey, the median number of full-time public health workers in medical institutions is 5, with 5 in tertiary hospitals, 3 in secondary hospitals, and 8 in primary and lower-level medical institutions. Among the 2,660 public health personnel surveyed, the median age was 40.0 years, 79.7% (2,119/2,660) were female, and 61.6% (1,639/2,660) held a bachelor’s degree. The survey of public health personnel indicates that 42.5% (1,131/2,660) are from nursing majors, while those from public health majors constitute only 17.5% (465/2,660). Primary and intermediate professional titles account for 45.6% (1,109/2,434) and 30.6% (745/2,434), respectively. There were statistically significant differences in the number, age, gender, educational background, profession, occupational category, and professional title of public health personnel across different levels of medical institutions, as illustrated in Table 2.

**Table 2:** Basic information on public health practitioners.

Characteristics	Hospitals of grade II or above	Hospitals of sub-grade II	Total	*p*-value*
Age, *M* (*IQR*)	43.0 (18.0)	39.0 (16.0)	40.0 (18.0)	*<0.001*
Gender				*0.017*
Male	190 (23.1)	351 (19.1)	541 (20.3)	
Female	631 (76.9)	1,488 (80.9)	2,119 (79.7)	
Education				
Junior college	156 (19.0)	756 (41.1)	912 (34.3)	*<0.001*
Bachelor	584 (71.1)	1,055 (57.4%)	1,639 (61.6)	
Master	77 (9.4)	28 (1.5%)	105 (3.9)	
Doctor	4 (0.5)	-	4 (0.2)	
Major				*<0.001*
Public health related	171 (20.8)	294 (16.0)	465 (17.5)	
Nursing	359 (43.7)	772 (42.0)	1,131 (42.5)	
Clinical medicine	146 (17.8)	441 (24.0)	587 (22.1)	
Other medicine related Majors	78 (9.5)	227 (12.3)	305 (11.5)	
Non-medical related majors	67 (8.2)	105 (5.7)	172 (6.5%)	
Practicing requirements				0.057
Acquired	766 (93.3)	1,674 (91.0)	2,440 (91.7)	
Not acquired	55 (6.7)	165 (9.0)	220 (8.3)	
Practice Type				*<0.001*
Physician	266 (42.1)	741 (47.3)	1,007 (45.8)	
Nurse	285 (45.1)	703 (44.9)	988 (44.9)	
Researcher	34 (5.4)	7 (0.4)	41 (1.9)	
Other medical related titles	32 (5.1)	90 (5.7)	122 (5.5)	
Non-medical related titles	15 (2.4)	26 (1.7)	41 (1.9)	
Grade of professional title				*<0.001*
Primary	251 (32.9)	858 (51.3)	1,109 (45.6)	
Middle	241 (31.6)	504 (30.1)	745 (30.6)	
Deputy senior	178 (23.4)	266 (15.9)	444 (18.2)	
Senior	92 (12.1)	44 (2.6)	136 (5.6)	
Full-time staff				0.323
Yes	720 (33.3)	1,637 (30.5)	2,357 (30.9)	
No	101 (66.7)	202 (69.5)	303 (69.1)	

*The *p*-values for continuous variables were derived using the Wilcoxon rank-sum test, while those for categorical variables were determined through the chi-squared (χ^2^) test.

*p-values that exhibit statistically significant differences are italicized.*

### The construction and function of public Health work organizations

A comprehensive survey of the organizational structure of public health initiatives within medical institutions reveals that 171 such facilities have established public health management committees. Specifically, 44 are tertiary hospitals, 22 are secondary hospitals, and 105 are primary or lower-level medical institutions. Notably, 67.3% (115 out of 171) of these committees are chaired by the institution’s president. Committee membership primarily includes departments related to public health, medicine, nursing, and hospital administration; however, only 22.8% of the medical institutions have integrated clinical departments into their committees (for detailed information refer to Supplementary Table S1.

### Awareness of public health responsibility of medical institutions

A total of 19,026 non-public health medical personnel participated in the survey regarding their perception of public health work, encompassing administrative, clinical, medical technology, and logistical support departments. The mean age of the participants was 36.7 years, with 72.9% being female and 68.0% holding a bachelor’s degree. Survey findings indicated that 99.2% of the respondents endorsed the notion that medical institutions should engage in public health activities; 86.5% advocated for the establishment of independent public health departments within these institutions; and 98.3% recognized the necessity of collaboration with disease control agencies for effective public health work in hospitals. The primary objections to public health involvement included: (1) the belief that medical institutions should prioritize patient care, leaving public health responsibilities to the Centers for Disease Control (CDC) and primary healthcare centers; (2) concerns over the additional workload and its impact on already busy schedules; (3) the potential disruption to clinical operations; and (4) limitations in available human resources. To assess the level of awareness concerning public health tasks, the survey focused on four key indicators: management of infectious disease outbreaks and associated information, disinfection and infection control, registration and reporting of deaths due to chronic non-communicable diseases, and institutional health emergency response. Results revealed that awareness levels across all four indicators exceeded 50%, with the highest rate observed for disinfection and infection control at 74.1% (14,097/19,026). The lowest awareness was noted for the registration of deaths from chronic non-communicable diseases at 57.4% (10,917/19,026). Awareness rates for the management of infectious disease outbreaks and health emergency response stood at 63.7% (12,121/19,026) and 63.4% (12,056/19,026), respectively.

### SWOT analysis of public health programs implementation

Following in-depth interviews with key informants, this paper systematically categorized the factors based on their frequency of mention and the interviewees’ prioritization of their significance.

The initial phase involves conducting a comprehensive analysis of the four key factors: strengths, weaknesses, opportunities, and threats (SWOT). Strengths and weaknesses pertain to internal conditions, encompassing elements such as human resources, financial capital, physical assets, technological infrastructure, available resources, and strategic thinking. These aspects can be influenced through internal initiatives, and appropriate internal assessment methodologies can be employed to accurately identify these attributes. Conversely, opportunities and threats are external environmental factors, which include governmental policies and regulations, societal norms, political landscapes, economic conditions, demographic structures, as well as market trends and competitive dynamics within industries. It is crucial to recognize that a SWOT analysis serves as an objective portrayal of the present state rather than an aspirational depiction. Subsequently, the second stage entails the construction of the SWOT matrix. This process involves gathering data on the status of various factors and organizing them into the matrix based on their urgency, impact, and significance. Finally, the third step is to formulate an action plan. Utilizing systems analysis techniques, the interplay between different environmental factors is analyzed to derive potential strategies, leading to the development of actionable solutions.

The countermeasures are detailed in Supplementary Table S2. SO countermeasures involve leveraging external opportunities while fully utilizing internal strengths to accelerate development, representing the optimal strategic combination. ST countermeasures aim to mitigate or avoid external threats while capitalizing on internal strengths. WO countermeasures focus on seizing external opportunities while addressing internal weaknesses. WT countermeasures entail overcoming internal weaknesses and safeguarding against external threats. Based on the analysis results from the SWOT matrix, it has been further elucidated which strengths, weaknesses, opportunities, and risks should be prioritized in the implementation of public health initiatives in hospitals. Rational recommendations have been formulated accordingly, as detailed in Table 3.

**Table 3:** SWOT analysis of public health programs implementation in public health department of medical institution.

Strength	The combination of clinical and public health services can more directly target the needs of patients and improve the prevention and treatment effect. In the process of diagnosis and treatment, clinicians can directly provide health education and promotion suggestions to patients to improve their health awareness and behavior. Hospitals, as the main providers of medical services, can conveniently collect and report patients’ health information and provide data support for public health decision-making. Hospitals generally have stronger human resources and medical equipment to better support the implementation of public health services. The hospital has authority in the diagnosis and treatment of diseases, and can more effectively intervene in the disease of key populations. The original public health service of the hospital provides a good foundation and experience for the new public health work.
Weakness	Public health work involves many aspects and the task is heavy, and the unreasonable incentive mechanism in the hospital may lead to low enthusiasm of staff. The shortage and structure of public health professionals can limit the quality and scope of public health services. Unclear coordination mechanisms between clinical and public health work can lead to duplication or omission of work, affecting work efficiency. The lack of financial support makes it difficult to carry out the work
Opportunity	National policy support. The standard public health service work content provides a clear direction for hospitals. The policy support of the local government has provided strong support for the construction of the public health department of the hospital. The integrated health service is conducive to the implementation of public health functions and the optimal allocation of resources.
Threat	Hospitals may face additional difficulties in funding implementation, affecting the implementation of public health services. Shortages of internal resources can limit the availability and effectiveness of public health services. Staffing constraints may limit the quality and scope of public health services.

## Discussion

The study revealed that 250 medical institutions have established public health-related departments, with 70.6% of these institutions setting up independent public health departments, and 93.8% of secondary and higher-level hospitals doing the same. The establishment of public health departments in medical institutions in Jiangsu Province is essentially complete (8). Regarding the timing of establishment, 65.3% of the public health departments were established after 2021, particularly in secondary and higher-level medical institutions (91.7%), and are predominantly located in public general hospitals in southern Jiangsu. This indicates that the establishment of public health departments in hospitals in our province began relatively late, with increased focus due to the outbreak of the novel coronavirus pneumonia. The median number of full-time public health workers is 5, with 5 at the tertiary level, 3 at the secondary level, and 1 at the primary level. While the number of staff generally meets the setting requirements, most are female, nursing professionals, lacking in public health expertise and senior technical titles, with significant differences observed across different levels. This highlights the urgent need for professionalization and rationalization of public health personnel and structures in medical institutions at all levels. Although the establishment of institutions is largely complete, 59.5% of public health departments lack dedicated funding for operations. The Healthy China initiative attempt to change the health system from disease treatment to public health prevention, emphasizing the importance of health prevention and promotion (9). Over the past decade, total health spending has increased from $1,090 per capita in 2008 to $4,700 per capita in 2019, an increase of 331% (10). Although the spending on specialized public health organizations increased in absolute terms, their share of total health spending fell from 8.58 percent in 2008 to 5.47 percent in 2019, as more spending went to hospitals (11). It is recommended that medical institutions enhance their focus and investment in public health initiatives, transcending the establishment of mere organizational structures. We should shift the hospital-centric funding systems to supporting prevention and primary care, which is the most cost-effective way to invest in health care (12, 13).

The study also found that most medical institutions have established a public health management committee to coordinate public health efforts within the institution, but only 22.8% of these institutions have integrated clinical-related business departments into the committee. As the frontline for disease detection and monitoring, departments such as outpatient, clinical, and laboratory services should be part of the three-tier network management system (14), serving as key members of the management committee and undertaking public health tasks such as registering and reporting public health emergencies, diagnosing and treating infectious diseases, managing hospital infection control, and monitoring and managing chronic non-communicable diseases. Currently, medical institutions shoulder over 10 public health responsibilities with only 5 full-time public health staff, leading to “one post, multiple responsibilities” and “one person, multiple roles,” with cross-departmental collaboration and occasional inter-departmental conflicts (15, 16). The public health department should serve as the office under the public health management committee, responsible for comprehensive management, organization, and coordination of public health work, as well as daily disease surveillance and reporting. Medical institutions should integrate the functions and business departments of medical care, nursing, and hospital infection control, enhancing centralized management and internal coordination. Responsibilities should be clearly delineated to ensure the effective fulfillment of public health management duties.

Moreover, different levels of medical institutions have distinct public health functions and scopes. For instance, tertiary hospitals typically focus on public health management within the hospital, whereas secondary community hospitals and most primary public medical institutions must engage with the community and provide a wide range of basic public health services. Therefore, a scientific list of public health responsibilities should be developed for different levels of medical institutions.

Surveys of non-public health department staff within medical institutions revealed that while most agree that medical institutions should undertake public health responsibilities, their understanding of these duties is frequently confined to disinfection and hospital infection control. There is limited awareness of other critical health management tasks, such as the management of chronic non-communicable diseases. Additionally, 98.3% of respondents believe that hospital public health work requires support from CDC institutions, suggesting that enhanced communication and cooperation between CDCs and medical institutions could improve the effectiveness of public health efforts. Collaboration between health care and public health is important for improving community health and is an opportunity to strengthen public health systems while staying focused on improving the public’s health (6, 17). One-on-one in-depth interviews indicated that medical institutions generally hold a positive attitude toward public health and the integration of medical and preventive services, though a minority of staff express reservations (18). The main concerns include: (a) the financial burden on medical institutions due to insufficient public health investment; (b) job burnout among medical staff from frequent responses to public health emergencies (19, 20); (c) insufficient public health awareness and weak sense of responsibility within hospitals; (d) shortages of public health personnel and inadequate staffing; (e) an imperfect system and insufficient integration of medical and preventive services with disease control institutions (21, 22). To address these issues, it is essential to strengthen policy support, clarify the primary responsibility for disease prevention and control in medical institutions, broaden financing channels for public health, and establish a long-term co-financing model involving both the government and individuals (23, 24). Medical institutions and regulatory bodies should prioritize the physical and mental well-being of medical staff, reduce resistance to public health work, and incentivize active participation to boost staff enthusiasm. Finally, education and training programs should be strengthened to promote the integration of medical and preventive services, clarify the public health responsibilities of medical institutions, and establish robust assessment and evaluation mechanisms, fostering deeper integration through personnel exchanges, information sharing, and resource pooling.

This study has some limitations. The investigation focused on a selection of medical institutions in Jiangsu Province, and further sample surveys are needed to verify the findings.

## Conclusion

From the perspective of public health services in contemporary medical institutions, this study conducted an in-depth survey on the current status of public health work and the development of public health departments in Jiangsu Province. The findings indicate that medical institutions in Jiangsu Province need to reinforce the allocation of public health personnel, refine the organizational structure and delineation of responsibilities for public health tasks, and enhance professional knowledge and skills to effectively fulfill their public health duties. Additionally, it is recommended to establish a continuous medical service model that integrates curative and preventive services.

## Data Availability

The original contributions presented in the study are included in the article/Supplementary material, further inquiries can be directed to the corresponding author.
