# Supplementary material for: Current status of public health awareness and roles within medical institutions in eastern China
Source: Front Public Health. 2025 Jan 14;13:1546507. doi: 10.3389/fpubh.2025.1546507 (PMC11772271; doi:10.3389/fpubh.2025.1546507)
Supplement: Supplementary file 1 [file Data_Sheet_1.pdf]

Table S1 Work contents of public health practitioners

Responsibilities	Cases	Proportion (%)
Prevention of infectious diseases	605	22.7
Chronic non-communicable disease management	714	26.8
Health emergency	338	12.7
Health education and promotion	496	18.6
Foodborne illness information reporting management	353	13.3
Occupational health and radiological protection management	352	13.2
Maternal and child health service management	784	29.5
Vaccination management	736	27.7
Others*	276	10.4

Note: *Others include management personnel, management of persons with severe mental disorders, basic public health projects, health examinations, health supervision and management, and school public health。

Table S2 The construction of SWOT matrix and the formulation of action plan

Factors	Strength	Weakness
Opportunity	Combination I: SO counterplan<br>Leverage strengths and take advantage of opportunities	Combination II: WO counterplan<br>Take advantage of opportunities and overcome weaknesses
Threat	Combination III: ST counterplan<br>Use strengths and avoid threats	Combination IV: WT counterplan<br>Reduce weaknesses and avoid threats
